# Pharmacological Modulation of Endotoxin-Induced Release of IL-26 in Human Primary Lung Fibroblasts

**DOI:** 10.3389/fphar.2019.00956

**Published:** 2019-08-30

**Authors:** Karlhans Fru Che, Jitong Sun, Anders Linden

**Affiliations:** ^1^Unit for Lung and Airway Research, Institute of Environmental Medicine, Karolinska Institutet, Stockholm, Sweden; ^2^Department of Immunology, Genetics and Pathology, Experimental and Clinical Oncology, Uppsala University, Uppsala, Sweden; ^3^Department of Respiratory Disease and Allergy, Karolinska University Hospital Solna, Stockholm, Sweden

**Keywords:** anticholinergic, beta-agonist, bronchodilator, glucocorticoid, IL-26, IL-8, fibroblast, MAP kinase

## Abstract

**Background:** Interleukin (IL)-26 is a neutrophil-mobilizing and bactericidal cytokine that is enhanced in human airways *in vivo* in response to endotoxin from Gram-negative bacteria. This cytokine is also enhanced in the airways during exacerbations of chronic obstructive pulmonary disease (COPD). Here, we investigated whether human primary lung fibroblasts (HLF) release IL-26 constitutively and in response to TLR4 stimulation by endotoxin and characterized the effects of bronchodilatory and anti-inflammatory drugs utilized in COPD.

**Methods:** The HLF were stimulated with different concentrations of endotoxin. Cells were also treated with different concentrations of bronchodilatory and anti-inflammatory drugs, with and without endotoxin stimulation. Cytokine protein concentrations were quantified in the cell-free conditioned media [enzyme-linked immunosorbent assay (ELISA)], and the phosphorylation levels of intracellular signaling molecules were determined (phosphoELISA).

**Results:** Whereas HLF displayed constitutive release of IL-26 into the conditioned medium, endotoxin markedly enhanced this release, as well as that of IL-6 and IL-8. This cytokine release was paralleled by increased phosphorylation of the intracellular signaling molecules NF-κB, c-Jun N-terminal kinase (JNK) 1-3, p38, and extracellular signal-regulated kinase (ERK) 1/2. The glucocorticoid hydrocortisone caused substantial inhibition of the endotoxin-induced release of IL-26, IL-6, and IL-8, an effect paralleled by a decrease of the phosphorylation of NF-κB, p38, and ERK1/2. The muscarinic receptor antagonist (MRA) tiotropium, but not aclidinium, caused minor inhibition of the endotoxin-induced release of IL-26 and IL-8, paralleled by a decreased phosphorylation of NF-κB. The β2-adrenoceptor agonist salbutamol caused modest inhibition of the endotoxin-induced release of IL-26 and IL-8, paralleled by a decreased phosphorylation of NF-κB, JNK1-3, and p38. Similar pharmacological effects were observed for the constitutive release of IL-26.

**Conclusions:** The HLF constitute an abundant source of IL-26 that may contribute to local host defense against Gram-negative bacteria. Among the tested drugs, the glucocorticoid displayed the most powerful inhibitory effect, affecting the NF-κB, p38, and ERK1/2 signaling pathways. Whether or not this inhibition of IL-26 contributes to an increased risk for local infections in COPD requires further evaluation.

## Introduction

Interleukin (IL)-26 is a dimeric 171-amino acid protein that belongs to the IL-10 family of cytokines (Donnelly et al., 2010). IL-26 exerts its actions on IL-10R2 and IL-20R1, a receptor complex that has been identified in both leukocytes and structural cells (Hor et al., 2004; Che et al., 2014). Cytokine signaling through this receptor complex activates the signal transducer and activator of transcription (STAT) 1 and STAT 3 (Hor et al., 2004).

IL-26 is involved in the pathogenic mechanisms of several chronic inflammatory diseases, including rheumatoid arthritis (Corvaisier et al., 2012), chronic hepatitis C (Miot et al., 2015), and Crohn’s disease (Dambacher et al., 2009). This cytokine exerts direct antibacterial effects on bacteria in a similar manner as antimicrobial peptides (AMP) (Meller et al., 2015). Interestingly, IL-26 is abundant in the airways, and local IL-26 concentrations are further enhanced following exposure to the TLR4-agonist endotoxin (Che et al., 2014). Moreover, IL-26 potentiates neutrophil mobilization and stimulates the release of pro-inflammatory cytokines in alveolar macrophages, thereby illustrating the involvement of IL-26 in innate host defense in the airways (Bao et al., 2017; Che et al., 2014). Furthermore, local IL-26 concentrations are enhanced in uncontrolled compared with controlled disease among children with asthma (Konradsen et al., 2016). The same is true in the case of airway morbidities among long-term smokers, including exacerbations of chronic obstructive pulmonary disease (COPD), chronic bronchitis, and local bacterial colonization (Che et al., 2018). To date, it has been shown that IL-26 is produced by alveolar macrophages, T-lymphocytes (Che et al., 2014), and bronchial epithelial cells in the airways (Che et al., 2017). The detection of IL-26 production in bronchial epithelial cells is of particular interest and may, in part, explain the abundance of IL-26 in the airways (Che et al., 2014; Che et al., 2017; Che et al., 2018). This abundance also warrants further investigation of additional structural cell types, including lung fibroblasts, with respect to their ability to release IL-26.

As with lining bronchial epithelial cells in the airways, lung fibroblasts are important in the maintenance of lung structural integrity (Phan, 2008). However, it is established that lung fibroblasts also respond to clinically important stimuli, such as bacteria, viruses, or noxious gases, leading to the production of archetypal neutrophil-mobilizing cytokines. Among these cytokines, IL-6 and IL-8 are known for their involvement in the asthma and COPD endotypes that display excessive neutrophil accumulation and endotypes that often respond poorly to common bronchodilatory and anti-inflammatory drugs (Rennard, 1999; Knight, 2001; Togo et al., 2008; Zhang et al., 2011; Sun et al., 2014). This background provides an additional argument for the investigation of the release of IL-26 in lung fibroblasts as well.

Bronchodilators provide vital relief of symptoms in patients suffering from asthma and COPD. In addition, the anti-inflammatory effects of glucocorticoids may reduce the risk of exacerbations associated with these airway disorders. Nevertheless, there is an unmet clinical need for more effective therapies against certain phenotypes of asthma and COPD (van der Velden, 1998; Barnes and Adcock, 2003; Rice et al., 2007; Zou et al., 2016). To develop such therapies, it is necessary to improve the understanding of fundamental pathogenic mechanisms behind these airway disorders. Moreover, it is of potential clinical value to characterize the pharmacological effects of common anti-inflammatory and bronchodilatory drugs on mechanisms of immune signaling *via* recently discovered cytokines (Thornton Snider et al., 2012; Ji et al., 2014; Lambrecht and Hammad, 2015; Berg and Wright, 2016). For these reasons, we have examined whether human primary lung fibroblasts (HLF) constitutively release IL-26 as well as IL-26 release in response to the TLR4-agonist endotoxin (lipopolysaccharide from Gram-negative bacteria). For comparison, we characterized the associated release of the archetypal neutrophil-mobilizing cytokines IL-6 and IL-8 in the HLF. We then examined the extent to which glucocorticoids (hydrocortisone) or bronchodilators (aclidinium, tiotropium, and salbutamol) modulate the release of IL-26, as well as that of IL-6 and IL-8.

## Materials and Methods

### Cell Culture Conditions

The HLF (isolated from normal healthy human lung parenchyma; Cell Applications, San Diego) were cultured in culture media (10% fetal bovine serum (FBS) in Dulbecco’s modified Eagle medium (DMEM) (Thermo Fisher Scientific, Stockholm), supplemented with antibiotics (penicillin/streptomycin 100 µg/ml; Thermo Fisher Scientific) until cells were semi-confluent. Cells were then starved (6 h in 3% FBS) before stimulation (18 h, 5% CO_2_, 37°C) with different concentrations of endotoxin (lipopolysaccharide 0.1–10 µg ml; Sigma Aldrich, Stockholm) and vehicle, to determine a sub-optimal concentration suitable for the priming of HLF. The HLF were subsequently pre-treated with a glucocorticoid (hydrocortisone 0.01–100 µM; Sigma Aldrich), an established muscarinic receptor antagonist (MRA) (tiotropium bromide 1–100 nM; Sigma Aldrich), a novel MRA (aclidinium bromide 0.1–10,000 mM; AstraZeneca, Mölndal, Sweden), or a beta_2_-adrenoceptor agonist (salbutamol 0.01–10 µM; Sigma Aldrich), followed by priming with a sub-maximally effective concentration of endotoxin (1 µg/ml) or its vehicle (24 h in 5% CO_2_, 37°C). Conditioned media were then harvested and centrifuged (1,500 rpm for 10 min) to remove cells and debris. The supernatant was then separated and frozen as cell-free conditioned media at −80°C for the quantification of cytokine concentrations. The adherent cells in the culture wells were lysed and stored at −80°C for the quantification of intracellular phosphorylation. Notably, as opposed to the short stimulation time (15–60 min) required to achieve maximum phosphorylation of these intracellular molecules, we, however, extended the endotoxin-stimulation time to 24 h for practical reasons in order to accommodate the drug-cell exposure time.

### Cytokine Quantification

*Interleukin-26*. The concentrations of IL-26 protein in cell-free conditioned media were quantified utilizing a commercial enzyme-linked immunosorbent assay (ELISA) according to the manufacturer’s instructions (Cusabio Biotech^®^, Newmarket Suffolk, CB87SY England). In brief, diluted samples and standards were added to the wells in duplicates and incubated (2 h, 37°C, 5% CO_2_). Biotin-conjugated detection antibody was added to the plate for 1 h, followed by incubation with avidin-conjugated horseradish peroxidase for 1 h. Tetramethylbenzidine (TMB) substrate and a stop solution were then added. The optical density (OD) was measured at 450 nm using a microplate reader (Model Spectra Max 250, Molecular Devices^™^, Sunnyvale, CA).

*Interleukin-6 and interleukin-8*. The concentrations of IL-6 and IL-8 protein in cell-free conditioned media were quantified utilizing commercial ELISA kits according to the manufacturer’s instructions (R&D Systems Europe Ltd, UK). In brief, high protein-binding ELISA plates (Nunc) were pre-coated (overnight at room temperature (RT)) with the respective IL-6 (2 µg/ml) and IL-8 (4 µg/ml) capture antibodies and blocked with 1% bovine serum albumin (BSA) (1 h, RT). The diluted samples and standards were added in duplicate and incubated for 2 h, at RT, followed by incubation (2 h, RT) with the secondary antibody (50 ng/ml for IL-6 and 20 ng/ml for IL-8). The TMB substrate and a stop solution were then added, and the OD was measured at 450 nm using a microplate reader.

### Quantification of Intracellular Phosphorylation

Intracellular phosphorylation/activation levels of nuclear factor kappa-light-chain-enhancer of activated B cells (NF-κB), the mitogen-activated protein (MAP) kinases (JNK1-3, p38, and ERK 1/2) were measured using PhosphoTracer ELISA according to the manufacturer’s instructions (Abcam^®^, Cambridge Science Park, UK). The cell lysates were thawed and transferred to the phosphoELISA plates in duplicate, and the specific capture and detection antibodies (at a ratio of 1:1) for the respective analytes were added and incubated (1 h on a shaker at 300 rpm, RT). The TMB substrate and stop solution were then added, and the OD was measured at 450 nm using a microplate reader.

### Statistical Analysis

Nonparametric descriptive and analytical statistics were applied to the study data (GraphPad Prism^®^ software). The Wilcoxon matched-pairs signed-rank test, Mann–Whitney test, or linear regression analyses were used, and *p* < 0.05 was considered statistically significant. *n* refers to the number of independent experiments (i.e., experiments on cells performed on separate days).

## Results

### Endotoxin-Induced Release of IL-26

**Figure 1:**
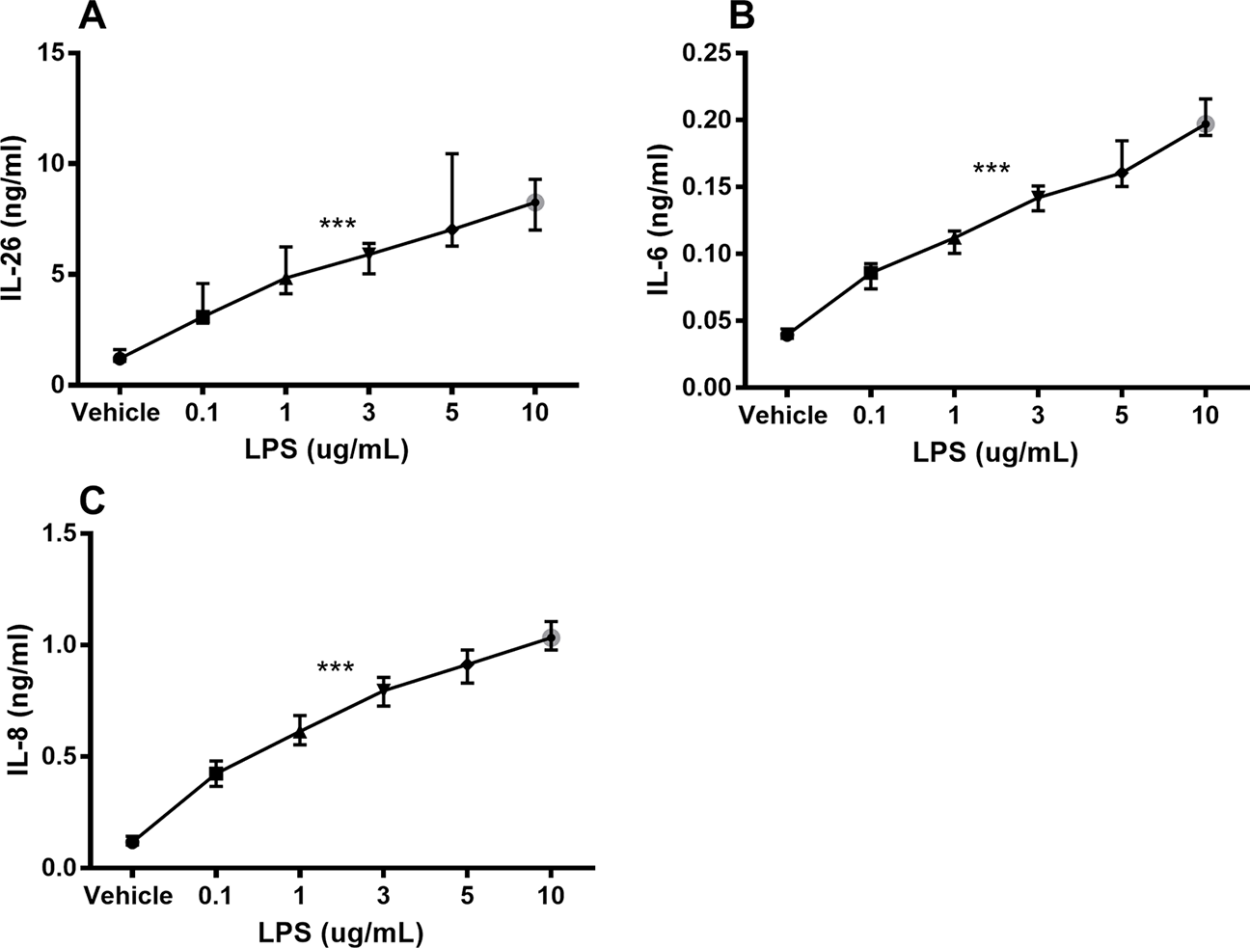
Endotoxin-induced release of IL-26, IL-6, and IL-8 protein in human primary lung fibroblasts. The cells were cultured *in vitro* and stimulated (24 h) with different concentrations of endotoxin (lipopolysaccharide, LPS), and the cytokine protein concentrations in cell-free conditioned media were quantified using ELISA. **(A)** IL-26 concentrations (*n* = 7), **(B)** IL-6 concentrations (*n* = 7), and **(C)** IL-8 concentrations (*n* = 7). The results are presented as median and range, and the *p*-values are according to the linear regression test. *p*-values < 0.05 were considered statistically significant. ***p ≤ 0.001.

Unstimulated HLF constitutively released IL-26 protein into conditioned media (**Figure 1A**). Moreover, stimulation with endotoxin markedly increased the IL-26 protein concentrations in a concentration-dependent manner (**Figure 1A**). We also observed a corresponding concentration-dependent release of IL-6 (**Figure 1B**) and IL-8 (**Figure 1C**) protein.

### Endotoxin-Induced Phosphorylation of NF-κB, JNK1-3, p38, and ERK1/2

**Figure 2:**
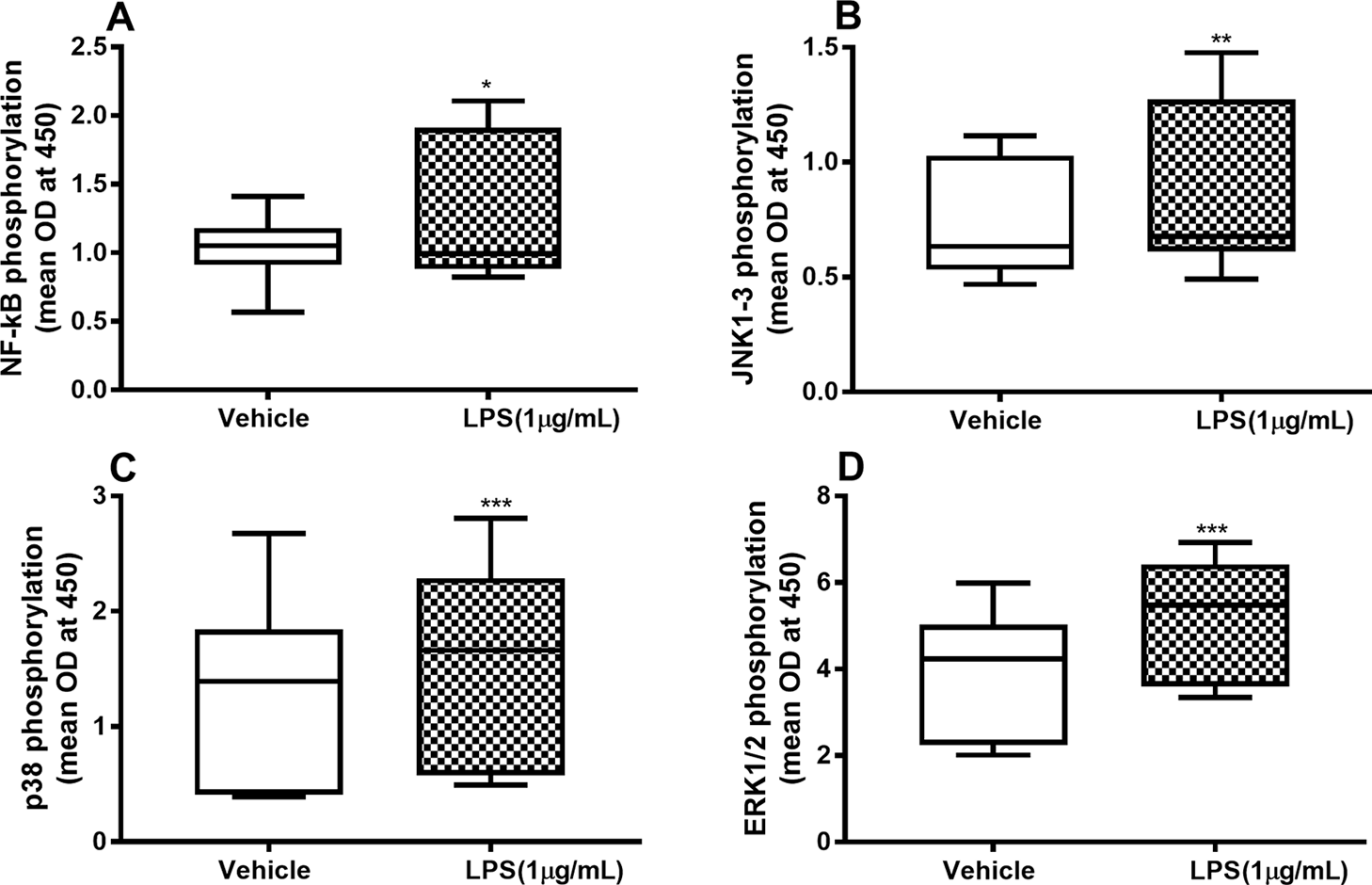
Endotoxin-induced phosphorylation of NF-κB, JNK1-3, p38, and ERK1/2 in primary human primary lung fibroblasts. The cells were cultured *in vitro* and stimulated with endotoxin (lipopolysaccharide, LPS) (1 µg/ml) for 24 h. The cells were lysed, and the levels of phosphorylated NF-κB, JNK1-3, p38, and ERK1/2 measured using phosphoELISA. Data were collected and presented as optical density (OD). **(A)** NF-κB (*n* = 14), **(B)** JNK1-3 (*n* = 14), **(C)** p38 (*n* = 14), and **(D)** ERK1/2 (*n* = 14). The results are presented as median and range, and the *p*-values are according to the Wilcoxon matched-pairs signed-rank test. *p-*values < 0.05 were considered statistically significant. *P < 0.05, **P < 0.005 and ***p ≤ 0.001.

To characterize the involvement of NF-κB and MAP kinases in the IL-26, IL-6, and IL-8 release process, we examined whether endotoxin triggers phosphorylation of these molecules and, moreover, whether the phosphorylation is maintained beyond 24 h. We found a sustained and statistically significant increase in the phosphorylation of NF-κB (**Figure 2A**), JNK1-3 (**Figure 2B**), p38 (**Figure 2C**), and ERK1/2 (**Figure 2D**) after 24-h exposure to endotoxin than in the control.

### Effects of Hydrocortisone on the Constitutive and Endotoxin-Induced Release of IL-26, IL-6, and IL-8

**Figure 3:**
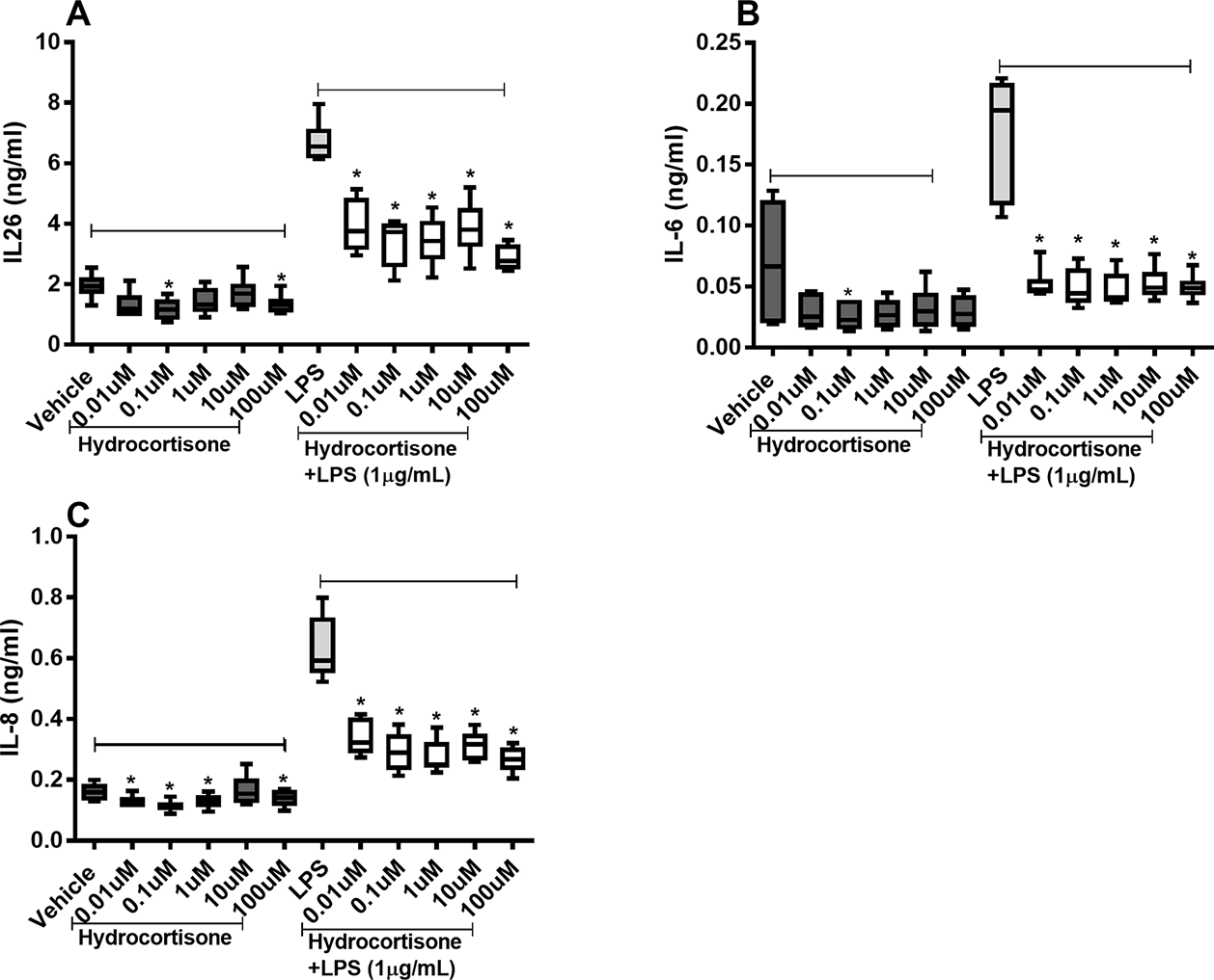
Hydrocortisone on the constitutive and endotoxin-induced release of IL-26, IL-6, and IL-8 in human primary lung fibroblasts. The cells were cultured *in vitro* and treated with different concentrations of hydrocortisone, with and without endotoxin (lipopolysaccharide, LPS) stimulation (1 µg/ml) for 24 h. The cytokine protein concentrations in cell-free conditioned media were quantified using ELISA. **(A)** IL-26 concentrations (*n* = 6), **(B)** IL-6 concentrations (*n* = 6), and **(C)** IL-8 concentrations (*n* = 6). The results are presented as median and range, and the *p*-values are according to the Wilcoxon matched-pairs signed-rank test. *p*-values < 0.05 were considered statistically significant. *P < 0.05.

Hydrocortisone (0.1–100 µM) caused substantial inhibition of constitutive IL-26 protein release as well as (0.01–100 µM) endotoxin-induced release of IL-26 in the HLF (**Figure 3A**). Likewise, hydrocortisone (0.1 µM) inhibited the constitutive release of IL-6 as well as (0.01–100 µM) endotoxin-induced release of IL-6 (**Figure 3B**). The same was true for the effect of hydrocortisone (0.01–100 µM) on the constitutive release of IL-8 and (0.01–100 µM) the endotoxin-induced release of IL-8 (**Figure 3C**).

### Effects of Hydrocortisone on the Constitutive and Endotoxin-Induced Phosphorylation of NF-κB, JNK1-3, p38, and ERK1/2

**Figure 4:**
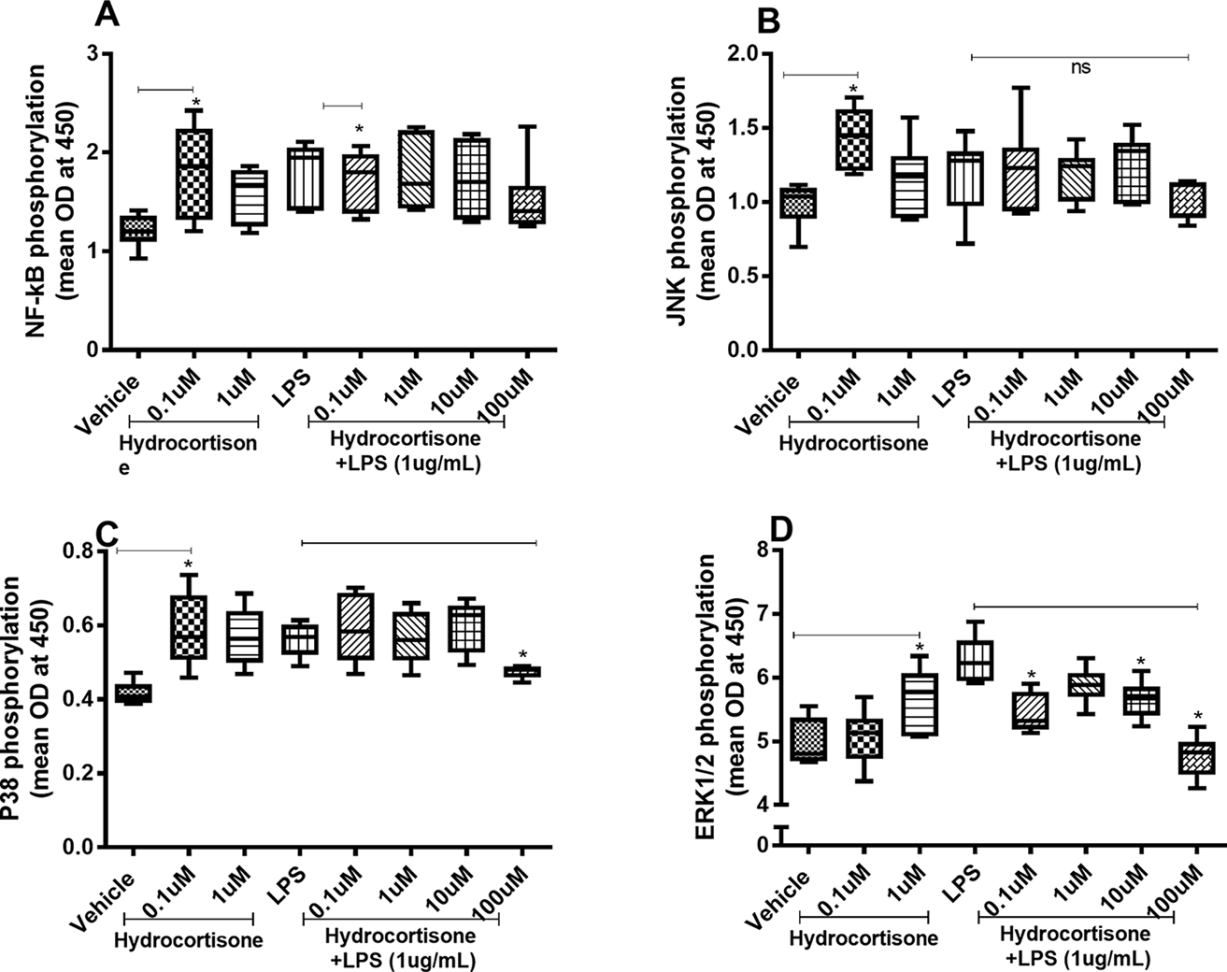
Hydrocortisone on the constitutive and endotoxin-induced phosphorylation of NF-κB, JNK1-3, p38, and ERK1/2 in human primary lung fibroblasts. The cells were cultured *in vitro* and treated with different concentrations of hydrocortisone, with and without endotoxin (lipopolysaccharide, LPS) stimulation (1 µg/ml) for 24 h. Cells were lysed, and the levels of phosphorylated NF-κB, JNK1-3, p38, and ERK1/2 were measured using phosphoELISA. Data were collected and presented as optical density (OD). **(A)** NF-κB (*n* = 6), **(B)** JNK1-3 (*n* = 6), **(C)** p38 (*n* = 5), and **(D)** ERK1/2 (*n* = 6). The results are presented as median and range, and the *p-*values are according to the Wilcoxon matched-pairs signed-rank test **(A**, **B**, and **D)** and Mann–Whitney test **(C)**. *p-*values < 0.05 were considered statistically significant. *P < 0.05; ns, nonsignificant.

When we investigated whether hydrocortisone alters the constitutive or endotoxin-induced phosphorylation of NF-κB, p38, JNK1-3, and ERK1/2, we found somewhat divergent effects. Hydrocortisone (0.1 or 1 µM), on its own, triggered increased phosphorylation of NF-κB (**Figure 4A**), JNK1-3 (**Figure 4B**), p38 (**Figure 4C**), and ERK1/2 (**Figure 4D**) in HLF. However, when we examined the effects of hydrocortisone on the endotoxin-induced phosphorylation of these molecules, hydrocortisone (at variable concentrations) inhibited the phosphorylation of NF-κB (**Figure 4A**), p38 (**Figure 4C**), and ERK1/2 (**Figure 4D**). However, hydrocortisone did not significantly alter the endotoxin-induced phosphorylation of JNK1-3 (**Figure 4B**).

### Effects of Tiotropium on the Constitutive and Endotoxin-Induced Release of IL-26, IL-6, and IL-8

**Figure 5:**
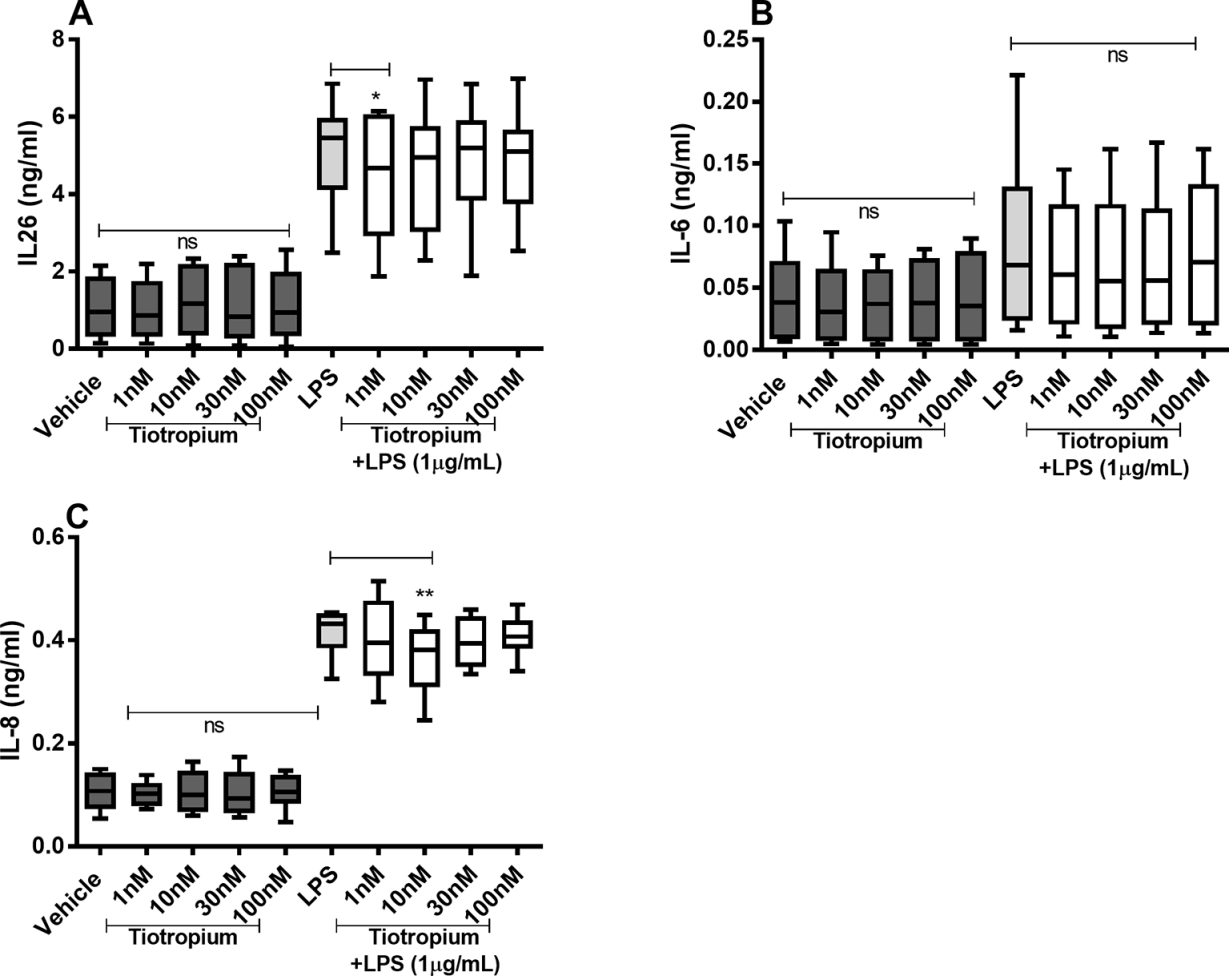
Tiotropium on the constitutive and endotoxin-induced release of IL-26, IL-6, and IL-8 in human primary lung fibroblasts. The cells were cultured *in vitro* and treated with different concentrations of tiotropium, with and without endotoxin (lipopolysaccharide, LPS) stimulation (1 µg/ml) for 24 h. The cytokine protein concentrations in cell-free conditioned media were quantified using ELISA. **(A)** IL-26 concentrations (*n* = 10). **(B)** IL-6 concentrations (*n* = 8). **(C)** IL-8 concentrations (*n* = 8). The results are presented as median and range, and the *p-*values according to the Wilcoxon matched-pairs signed-rank test. *p*-values < 0.05 were considered statistically significant. **P < 0.005; ns, nonsignificant.

Tiotropium did not cause any substantial effects on the constitutive release of IL-26, IL-6, and IL-8 proteins (**Figures 5A–C**) in the HLF. However, tiotropium (1 or 10 nM) caused a modest inhibition of the endotoxin-induced release of IL-26 (**Figure 5A**) and IL-8 (**Figure 5C**). In contrast, we found no reproducible effects of tiotropium on the endotoxin-induced release of IL-6 (**Figure 5B**).

### Effects of Tiotropium on the Endotoxin-Induced Phosphorylation of NF-κB, JNK1-3, p38, and ERK1/2

**Figure 6:**
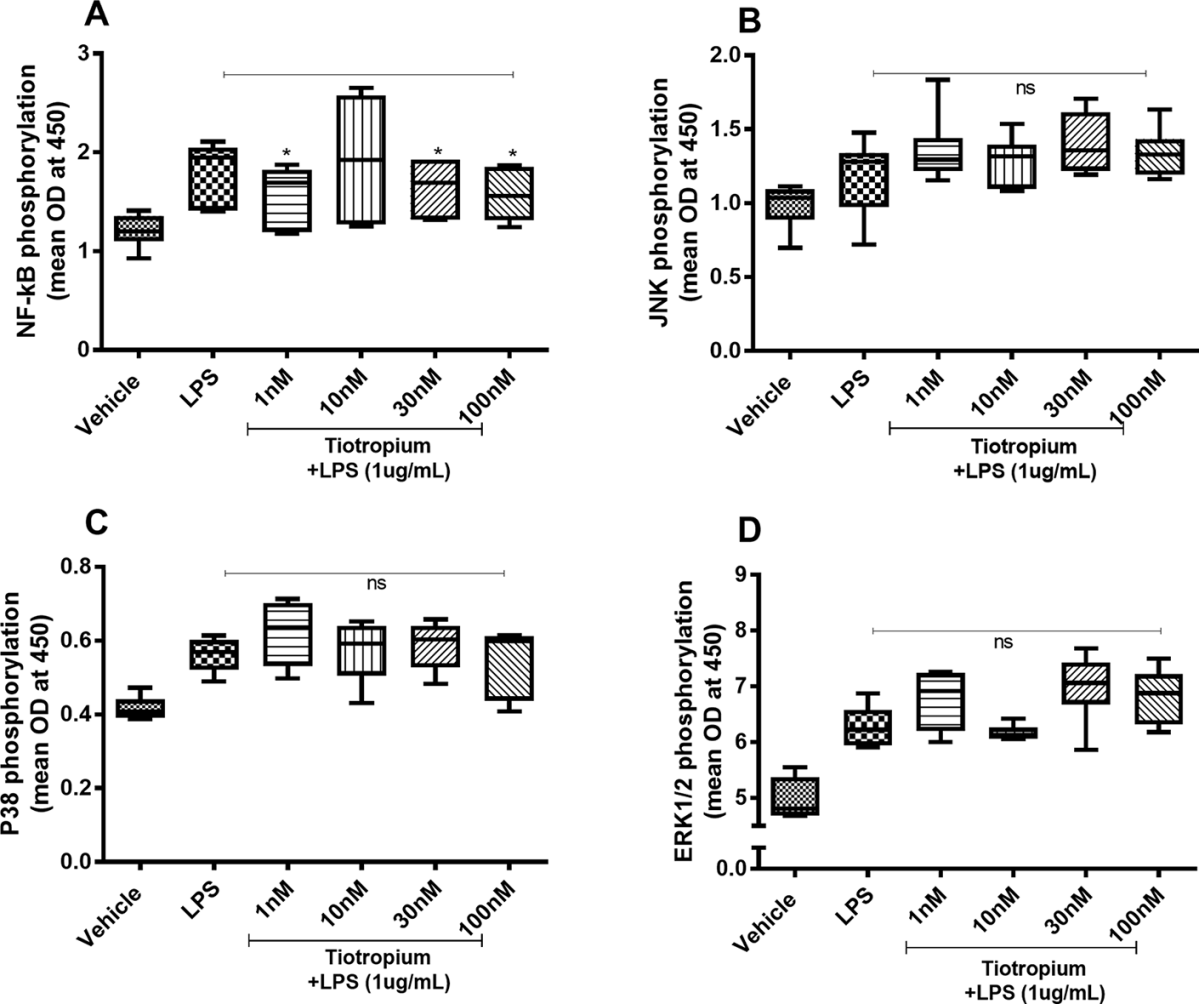
Tiotropium on the endotoxin-induced phosphorylation of NF-κB, JNK1-3, p38, and ERK1/2 in human primary lung fibroblasts. The cells were cultured *in vitro* and treated with different concentrations of tiotropium, with and without endotoxin (lipopolysaccharide, LPS) stimulation (1 µg/ml) for 24 h. Cells were lysed, and the levels of phosphorylated NF-κB, JNK1-3, p38, and ERK1/2 were measured using phosphoELISA. Data were collected and presented as optical density (OD). **(A)** NF-κB (*n* = 6), **(B)** JNK1-3 (*n* = 6), (C) p38 (*n* = 5), and **(D)** ERK1/2 (*n* = 6). The results are presented as median and range, and the *p-*values are according to the Wilcoxon matched-pairs signed-rank test **(A**, **B**, and **D)** and Mann–Whitney test **(C)**. *p-*values < 0.05 were considered statistically significant. *P < 0.05; ns, nonsignificant.

Because tiotropium exerted effects on the endotoxin-induced release of IL-26 and IL-8 only, we investigated the effects of this MRA on the endotoxin-induced phosphorylation of NF-κB, JNK1-3, p38, and ERK1/2 only. Tiotropium (1–100 nM) inhibited the endotoxin-induced phosphorylation of NF-κB (**Figure 6A**) but not that of JNK1-3 (**Figure 6B**), p38 (**Figure 6C**), or ERK1/2 (**Figure 6D**).

### Effects of Aclidinium on the Constitutive and Endotoxin-Induced Release of IL-26, IL-6, and IL-8

**Figure 7:**
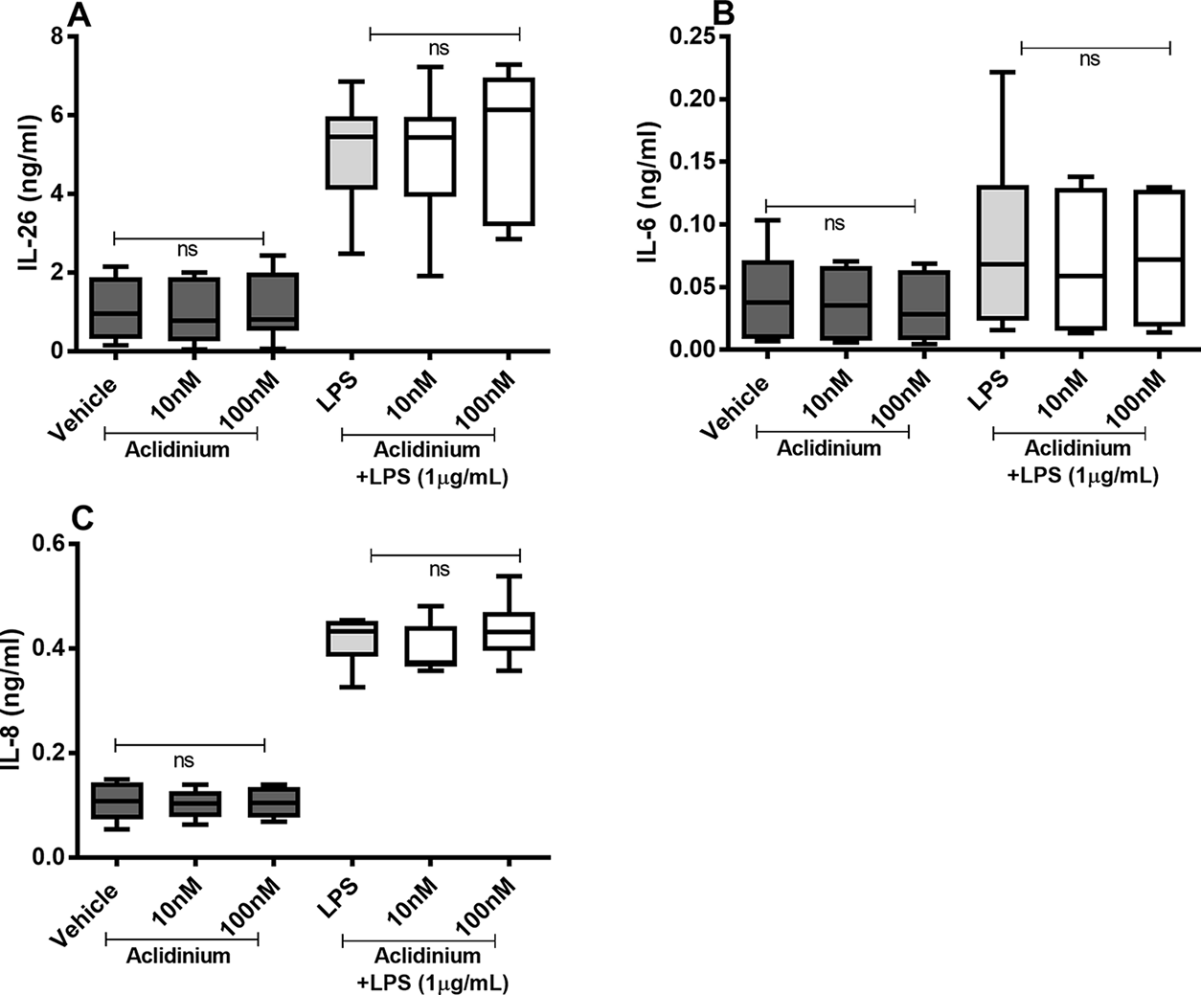
Aclidinium on the constitutive and endotoxin-induced release of IL-26, IL-6, and IL-8 on primary human primary lung fibroblasts. The cells were cultured *in vitro* and treated with different concentrations of aclidinium, with and without endotoxin (lipopolysaccharide, LPS) stimulation (1 µg/ml) for 24 h. The cytokine protein concentrations in cell-free conditioned media were quantified using ELISA. **(A)** IL-26 concentrations (*n* = 10). **(B)** IL-6 concentrations (*n* = 8). **(C)** IL-8 concentrations (*n* = 8). The results are presented as median and range, and the *p*-values are according to the Wilcoxon matched-pairs signed-rank test. *p*-values < 0.05 were considered statistically significant. ns, nonsignificant.

There were no reproducible effects of aclidinium on the constitutive or endotoxin-induced release of IL-26, IL-6, or IL-8 protein in the HLF (**Figures 7A–C**). Notably, when we tested lower concentrations of aclidinium (0.1 and 1 nM) (**Supplementary Figure 1**) and higher concentrations (1,000 and 10,000 nM) (**Supplementary Figure 2**), we obtained no effects of aclidinium on the release of IL-26, IL-6, and IL-8 proteins. Because of this, we did not perform any investigations of NF-κB, p38, JNK1-3, or ERK1/2 phosphorylation in this context.

### Effects of Salbutamol on the Constitutive and Endotoxin-Induced Release of IL-26, IL-6, and IL-8

**Figure 8:**
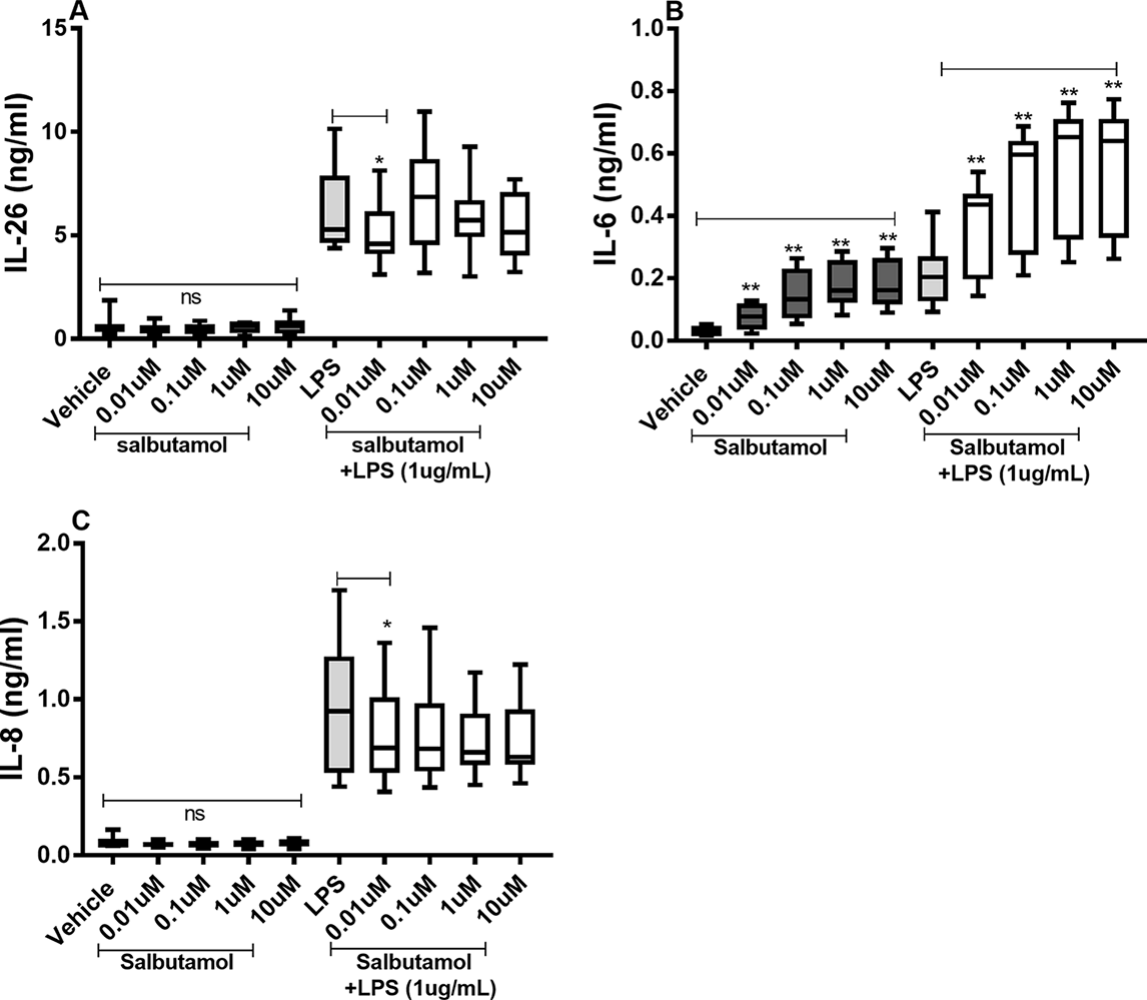
Salbutamol on the constitutive and endotoxin-induced release of IL-26, IL-6, and IL-8 in human primary lung fibroblasts. The cells were cultured *in vitro* and treated with different concentrations of salbutamol, with and without endotoxin (lipopolysaccharide, LPS) stimulation (1 µg/ml) for 24 h. The cytokine protein concentrations in cell-free conditioned media were quantified using ELISA. **(A)** IL-26 protein concentrations (*n* = 9), **(B)** IL-6 protein concentrations (*n* = 9), and **(C)** IL-8 protein concentrations (*n* = 9). The results are presented as median and range, and the *p*-values are according to the Wilcoxon matched-pairs signed-rank test (*n* = 6). *p*-values < 0.05 were considered statistically significant. **P < 0.005; ns, nonsignificant.

There were no reproducible effects of salbutamol on the constitutive release of IL-26 (**Figure 8A**) or IL-8 (**Figure 8C**) in the HLF. However, we did observe that salbutamol (0.01 µM) decreased the endotoxin-induced release of IL-26 (**Figure 8A**) and IL-8 (**Figure 8C**). In contrast, salbutamol (0.01–10 µM) increased the constitutive and endotoxin-induced release of IL-6 in the HLF (**Figure 8B**).

### Effects of Salbutamol on the Constitutive and Endotoxin-Induced Phosphorylation of NF-κB, JNK1-3, p38, and ERK1/2

**Figure 9:**
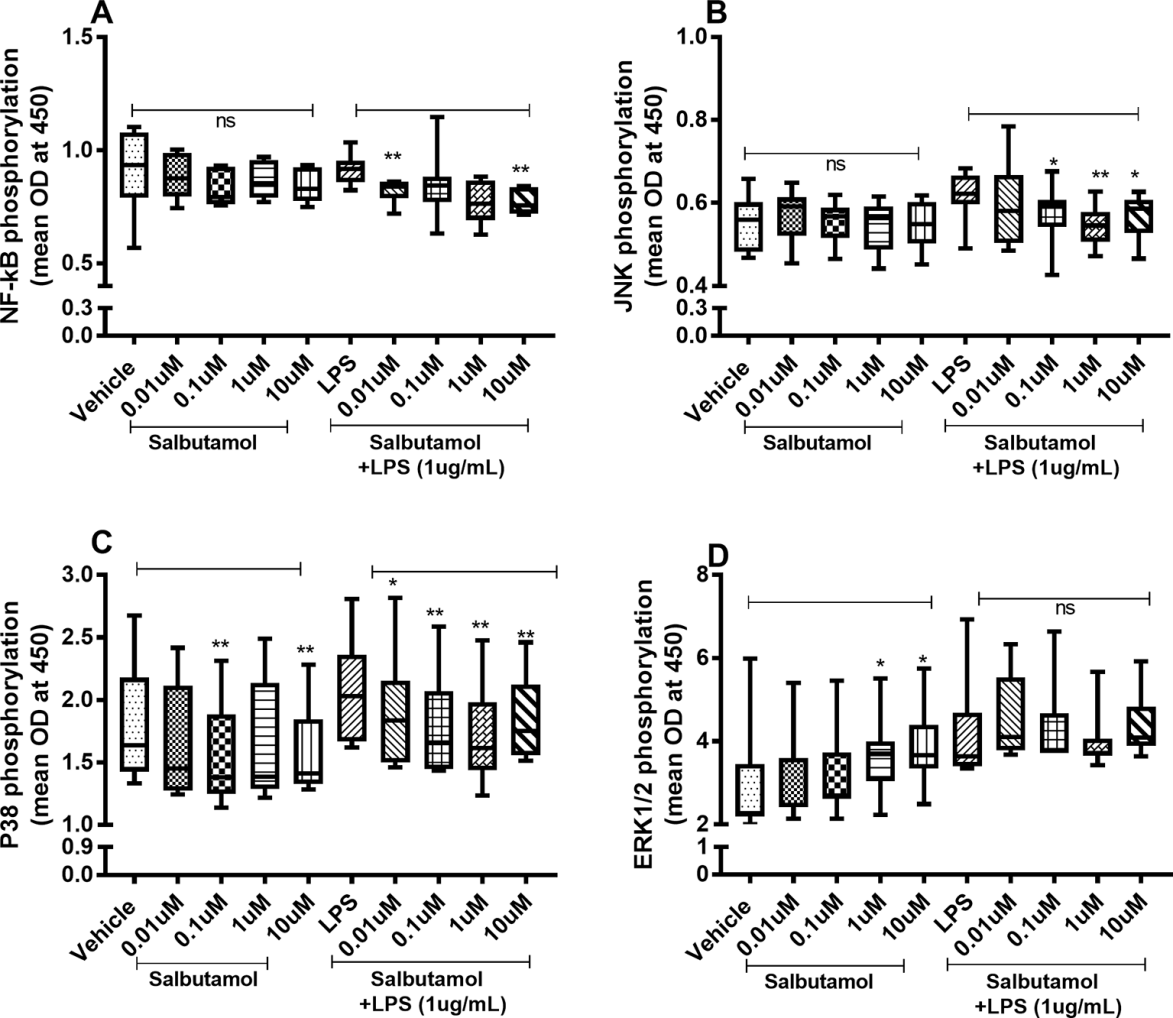
Salbutamol on the constitutive and endotoxin-induced phosphorylation of NF-κB, JNK1-3, p38, and ERK 1/2 in human primary lung fibroblasts. The cells were cultured *in vitro* and treated with different concentrations of hydrocortisone, with and without endotoxin (lipopolysaccharide, LPS) stimulation (1 µg/ml) for 24 h. Cells were lysed, and the levels of phosphorylated NF-κB, JNK1-3, p38, and ERK1/2 were measured using phosphoELISA. Data were collected and presented as optical density (OD). **(A)** NF-κB (*n* = 8), **(B)** JNK1-3 (*n* = 8), **(C)** p38 (*n* = 8), and **(D)** ERK1/2 (*n* = 8). The results are presented as median and range, and the *p*-values are according to the Wilcoxon matched-pairs signed-rank test. *p*-values < 0.05 were considered statistically significant. **P < 0.005; ns, nonsignificant.

There were no reproducible effects of salbutamol on the constitutive phosphorylation of NF-κB (**Figure 9A**) or JNK1-3 (**Figure 9B**). However, salbutamol (0.1, 1, and/or 10 µM) decreased the endotoxin-induced phosphorylation of NF-κB (**Figure 9A**) and JNK1-3 (**Figure 9B**). Moreover, when we examined p38, we found that salbutamol (0.01, 0.1, 1, and/or 10 µM) decreased the constitutive as well as endotoxin-induced phosphorylation of these proteins (**Figure 9C**). In contrast, salbutamol (1 and 10 µM) enhanced the constitutive phosphorylation of ERK1/2 but exerted no reproducible effect on endotoxin-induced phosphorylation (**Figure 9D**).

## Discussion

In this experimental study, we found that HLF constitutively releases IL-26 and that this release is substantially increased in response to the TLR4-agonist endotoxin (Raetz and Whitfield, 2002). As expected, the HLF also released the neutrophil-mobilizing cytokines IL-6 and IL-8, constitutively and in response to endotoxin (Zhang et al., 2011). Thus, given their abundance in the lungs, HLF may collectively constitute an important source of IL-26 in human airways that are involved in the innate immune response to Gram-negative bacteria (Che et al., 2014). This principal finding adds to previous evidence that IL-26 can be produced by multiple cellular sources in the airways (Che et al., 2014; Che et al., 2017; Che et al., 2018). Taken together, these findings could explain the abundance of IL-26 observed in previous *in vivo* studies in humans (Che et al., 2014; Che et al., 2018). This finding also supports our previous data, demonstrating that IL-26 also plays a role in the clinical setting, where Gram-negative bacteria constitute an important pathogenic stimulus for exacerbations of diseases such as pneumonia and COPD (Che et al., 2014; Che et al., 2018).

Upon examining intracellular signaling pathways that have previously been linked to the production of IL-26 (Che et al., 2017), we found that stimulation of HLF with endotoxin causes increased and sustained phosphorylation of NF-κB and the MAP kinases p38, JNK1-3, and ERK1/2 for at least 24 h. This finding is critical given that the phosphorylation of these molecules is known to peak between 15 and 60 min after stimulation. Moreover, increased phosphorylation of the very same intracellular signaling molecules, matched by increased release of IL-26, was demonstrated in a recent study on human primary bronchial epithelial cells stimulated with the TLR3 agonists poly-IC. In that study, the selective inhibition of these intracellular signaling compounds curtailed the release of IL-26 protein (Che et al., 2017). Thus, since the endotoxin-induced phosphorylation of NF-κB and p38, JNK1-3, and ERK1/2 in HLF is matched by an increased release of IL-26, our current findings are consistent with these intracellular signaling pathways being involved in the endotoxin-induced release of IL-26 in HLF. Such is also the case for the associated release of the two “classical” neutrophil-mobilizing cytokines IL-6 and IL-8.

We found that the glucocorticoid, hydrocortisone, caused a powerful inhibition of the endotoxin-induced release of IL-26 protein, as well as that of IL-6 and IL-8 proteins. These effects of hydrocortisone were paralleled by decreased intracellular phosphorylation of NF-κB, p38, and ERK1/2, and not JNK1 3. Our observations strengthen the argument that NF-κB, p38, and ERK1/2 are involved in the signaling pathways responsible for the release of IL-26, as well as that of IL-6 and IL-8 in HLF, and that glucocorticoids regulate these pathways. Notably, it is known that glucocorticoid receptors (GRs) are present in fibroblasts (Pratt, 1978; van der Velden, 1998; Kadmiel and Cidlowski, 2013) and glucocorticoid–GR complexes are known to modulate inflammatory responses through interactions with NF-κB (Sousa et al., 1993; van der Velden, 1998) and MAP kinases (Kassel et al., 2001; De Bosscher et al., 2003; Smoak and Cidlowski, 2004). Given our results, this seems evident in the presence of TLR4 stimulation. Unexpectedly, we observed that although hydrocortisone decreased the constitutive release of IL-26, IL-6, and IL-8, this glucocorticoid did increase constitutive phosphorylation of NF-κB, JNK1-3, p38, and ERK1/2 in the HLF. This suggests that in the absence of TLR4 stimulation, the glucocorticoid employs alternative pathways leading to inhibited cytokine production. An interesting example of this is that endotoxin increases GR expression in murine macrophages (Salkowski and Vogel, 1992; van der Velden, 1998), potentially influencing signaling by glucocorticoids. Moreover, glucocorticoids may induce anti-inflammatory responses by inducing increased expression of critical mediators in immune signaling, such MKP-1 and secretory leukocyte protease inhibitor (Young et al., 1999; Abbinante-Nissen et al., 1995; Kassel et al., 2001; Dandona et al., 2014).

We found that the MRA, tiotropium, caused minor inhibition of the endotoxin-induced release of IL-26 and IL-8 protein, in a similar manner as with the phosphorylation of NF-κB caused by TLR4 stimulation. However, this was not the case for the MAP kinases JNK1-3, p38, and ERK1/2. These findings imply that in addition to antagonizing acetylcholine, tiotropium may also induce certain anti-inflammatory effects through inhibition of NF-κB, an effect that may account for the decrease in the release of IL-26 and IL-8 caused by TLR4 stimulation. Moreover, previous studies have shown that lung fibroblasts express mRNA for muscarinic (M) 1, M2, and M3 receptors and that treatment with tiotropium inhibits acetylcholine-induced proliferation of these cells, thereby illustrating a functional inhibitory effect on fibroblasts that can be exerted by MRAs (Pieper et al., 2007).

We also examined the MRA, aclidinium (Moulton and Fryer, 2011), and found that this drug affected neither the constitutive nor the endotoxin-induced release of IL-26, IL-6, or IL-8 protein. Although aclidinium acts in the same principal manner as tiotropium, by antagonizing the effects of acetylcholine on M1, M2, and M3 receptors (Moulton and Fryer, 2011; Zou et al., 2016), there are certain known pharmacological differences between these two drugs (Moulton and Fryer, 2011; Zou et al., 2016). For example, aclidinium dissociates faster from the M receptors than does tiotropium (Moulton and Fryer, 2011; Zou et al., 2016). In addition, aclidinium is more rapidly metabolized in plasma than is tiotropium (Moulton and Fryer, 2011; Zou et al., 2016). These differences may explain the lack of an effect induced by aclidinium on the release of IL-26 caused by TLR4 stimulation, compared with that of tiotropium. Our findings are compatible with tiotropium exerting additional effects on the innate arm of host defense that cannot be mimicked by aclidinium.

Finally, we examined the effects of the short-acting β_2_-adrenoceptor agonist salbutamol on the TLR4-stimulated HLF and found that it caused a modest inhibition of IL-26 and IL-8 release. On the contrary, salbutamol clearly increased the constitutive as well as endotoxin-induced release of IL-6, which is consistent with previous studies (Pickholtz et al., 2011). Moreover, when we examined the phosphorylation levels of NF-κB, JNK1-3, p38, and ERK1/2, we found that during TLR4 stimulation, salbutamol decreased the phosphorylation of NF-κB, JNK1-3, and p38, whereas the phosphorylation levels of ERK1/2 were increased in unstimulated cells (i.e., in the absence of endotoxin). The observed decrease in the endotoxin-induced phosphorylation of NF-κB, JNK1-3, and p38, as well as the release of IL-26 and IL-8, suggests that salbutamol exerts a modest anti-inflammatory action on the HLF. It is also worth noting that the salbutamol-induced release of IL-6 may also reflect anti-inflammatory responses, given that IL-6 can act as an anti-inflammatory cytokine under certain conditions (Scheller et al., 2011). However, the salbutamol-induced release of IL-6, which is paralleled by constitutive phosphorylation of ERK1/2, could represent a pro-inflammatory effect of IL-6, as has been previously reported (Tan et al., 2007; Yang et al., 2010; Scheller et al., 2011). The latter is of particular importance since β2-adrenoceptor agonists can induce IL-6 production through the ERK1/2, in a cAMP-independent manner (Tan et al., 2007; Yang et al., 2010). Moreover, previous studies indicate that β2-adrenoceptor stimulation may lead to either anti-inflammatory or pro-inflammatory effects, depending on the particular circumstances (Maris et al., 2005; Tan et al., 2007).

**Figure 10:**
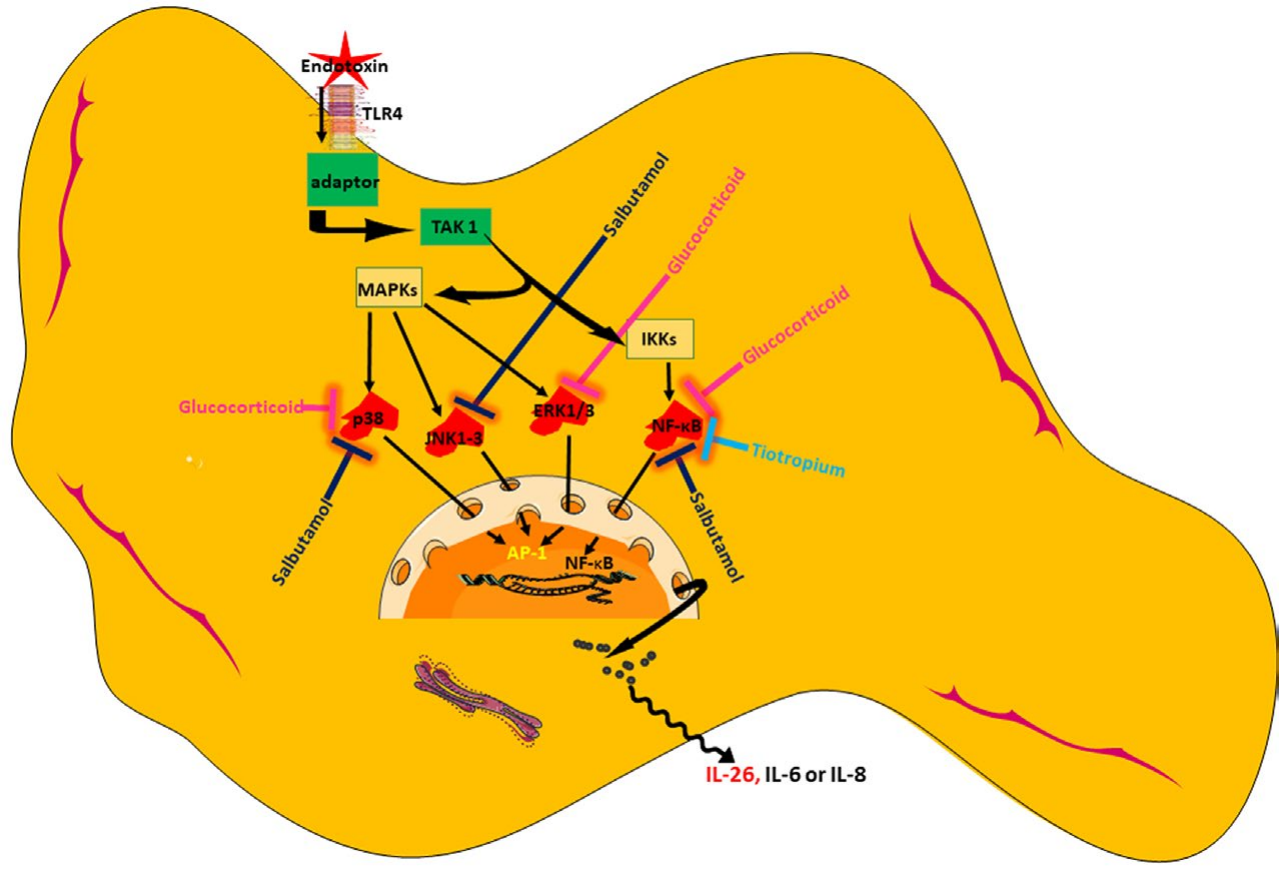
Schematic overview of endotoxin-induced release of IL-26 in human lung fibroblasts and the pharmacological effects caused by treatment with a glucocorticoid (hydrocortisone), a beta_2_-adrenoceptor agonist (salbutamol), and muscarinic receptor antagonist (tiotropium).

In conclusion, we present original evidence that HLF constitutes a potentially important source of IL-26, a cytokine with AMP-like features that is involved in innate host defense in the airways. We show that the release of IL-26 is constitutive and can be further enhanced by the TLR4-agonist endotoxin from Gram-negative bacteria. Among the tested pharmacological agents, a glucocorticoid distinguishes itself as the most effective in inhibiting IL-26 release, presumably by acting through NF-κB and MAP kinases. Moreover, one but not another MRA possesses modest inhibitory properties on the release of IL-26, presumably by acting through NF-κB. Finally, a short-acting β2-adrenoceptor agonist may also inhibit endotoxin-induced release of IL-26, presumably through NF-κB and MAP kinases (please see **Figure 10** showing a schematic overview). Thus, our current results introduce several strategies to modulate IL-26, with or without simultaneous activation of the innate arm of host defense, which are of potential utility in the treatment of diseases such as asthma and COPD. Clinical studies will be needed to address whether or not pharmacological inhibition of IL-26 has clinical benefits.

## Data Availability

All data generated or analyzed during this study are included in this published article (and its supplementary information files).

## Author Contributions

KC and AL initiated and designed the experiments. KC and JS performed the experiments. KC and AL wrote the manuscript. All authors read and approved the final manuscript.

## Funding

This study was sponsored by the Swedish Heart-Lung Foundation (HLF No. 20150303), the Swedish Research Council (VR No. 2016-01563), the Stockholm City Council (ALF No. 20140309), and AstraZeneca. The sponsors played no role in the design of the study and collection, analysis, and interpretation of data.

## Conflict of Interest Statement

The authors declare that the research was conducted in the absence of any commercial or financial relationships that could be construed as a potential conflict of interest.

## Supplementary Material

Supplementary Figure 1 Effect of low concentrations of aclidinium on the constitutive and endotoxin-induced release of IL-26, IL-6, and IL-8 in human primary lung fibroblasts. The cells were cultured *in vitro* and treated with different concentrations of aclidinium, with and without endotoxin (lipopolysaccharide, LPS) stimulation (1 µg/mL) for 24 h. The cytokine protein concentrations in cell-free conditioned media were quantified using ELISA. **(A)** IL-26 concentrations (*n* = 6). **(B)** IL-6 concentrations (*n* = 4). **(C)** IL-8 concentrations (*n* = 4). The results are presented as median and range, and the *p*-values are according to the Mann–Whitney test. *p*-values < 0.05 were considered statistically significant.

Supplementary Figure 2 Effect of high concentrations of aclidinium on the constitutive and endotoxin-induced release of IL-26, IL-6, and IL-8 in human primary lung fibroblasts. The cells were cultured *in vitro* and treated with different concentrations of aclidinium with and without endotoxin (lipopolysaccharide, LPS) stimulation (1 µg/mL) for 24 h. The cytokine protein concentrations in cell-free conditioned media were quantified using ELISA. **(A)** IL-26 concentrations (*n* = 4). **(B)** IL-6 concentrations (*n* = 4). **(C)** IL-8 concentrations (*n* = 4). The results are presented as median and range, and the *p-*values are according to the Mann–Whitney test. *p-*values < 0.05 were considered statistically significant.
