# Supplementary figures and images for: Pharmacological Modulation of Endotoxin-Induced Release of IL-26 in Human Primary Lung Fibroblasts
Source: Front Pharmacol. 2019 Aug 30;10:956. doi: 10.3389/fphar.2019.00956 (PMC6729122; doi:10.3389/fphar.2019.00956)

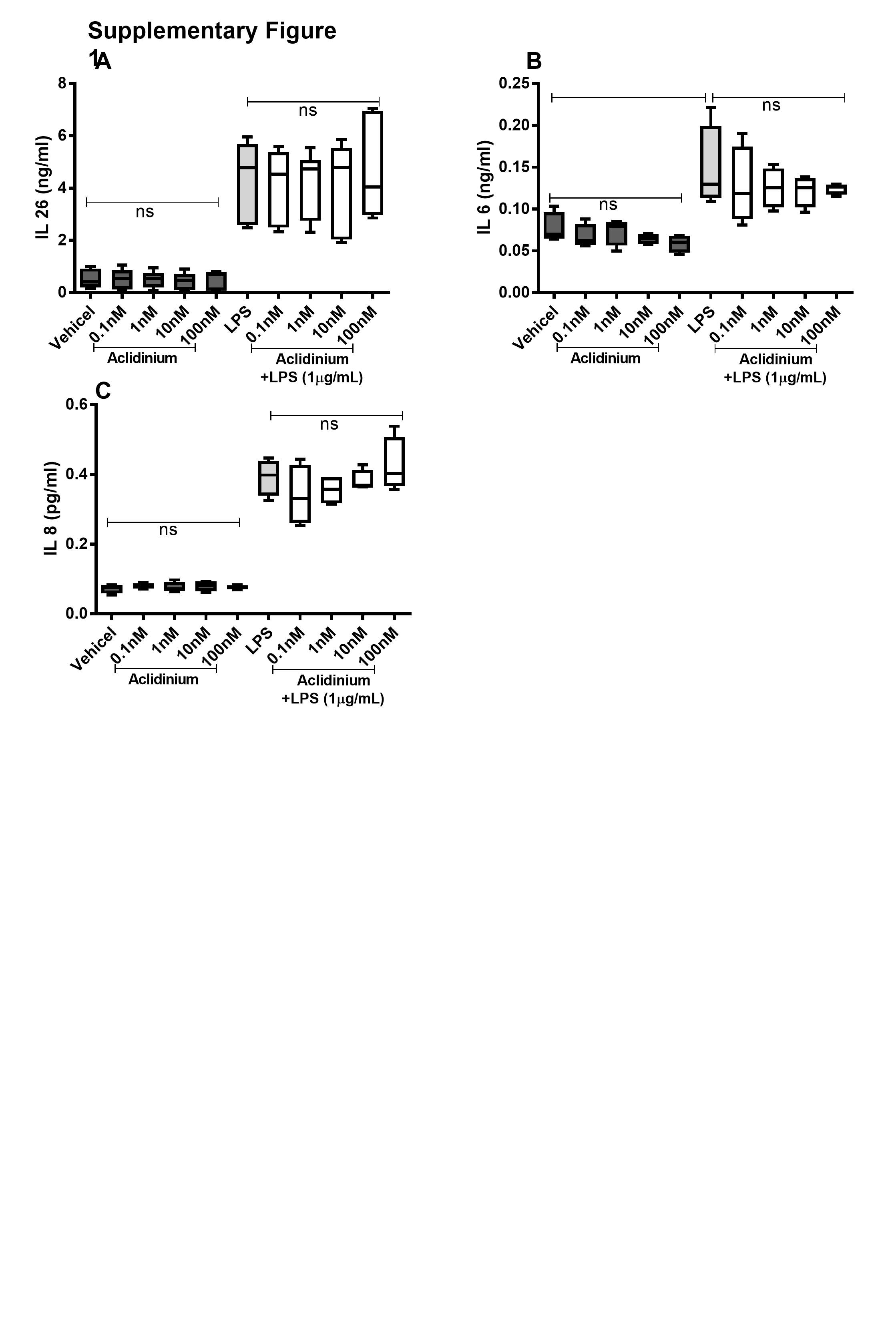

Supplement: Supplementary Figure 1 — Effect of low concentrations of aclidinium on the constitutive and endotoxin-induced release of IL-26, IL-6, and IL-8 in human primary lung fibroblasts. The cells were cultured in vitro and treated with different concentrations of aclidinium, with and without endotoxin (lipopolysaccharide, LPS) stimulation (1 µg/mL) for 24 h. The cytokine protein concentrations in cell-free conditioned media were quantified using ELISA. (A) IL-26 concentrations (n = 6). (B) IL-6 concentrations (n = 4). (C) IL-8 concentrations (n = 4). The results are presented as median and range, and the p-values are according to the Mann–Whitney test. p-values < 0.05 were considered statistically significant. [file Image_1.tif]

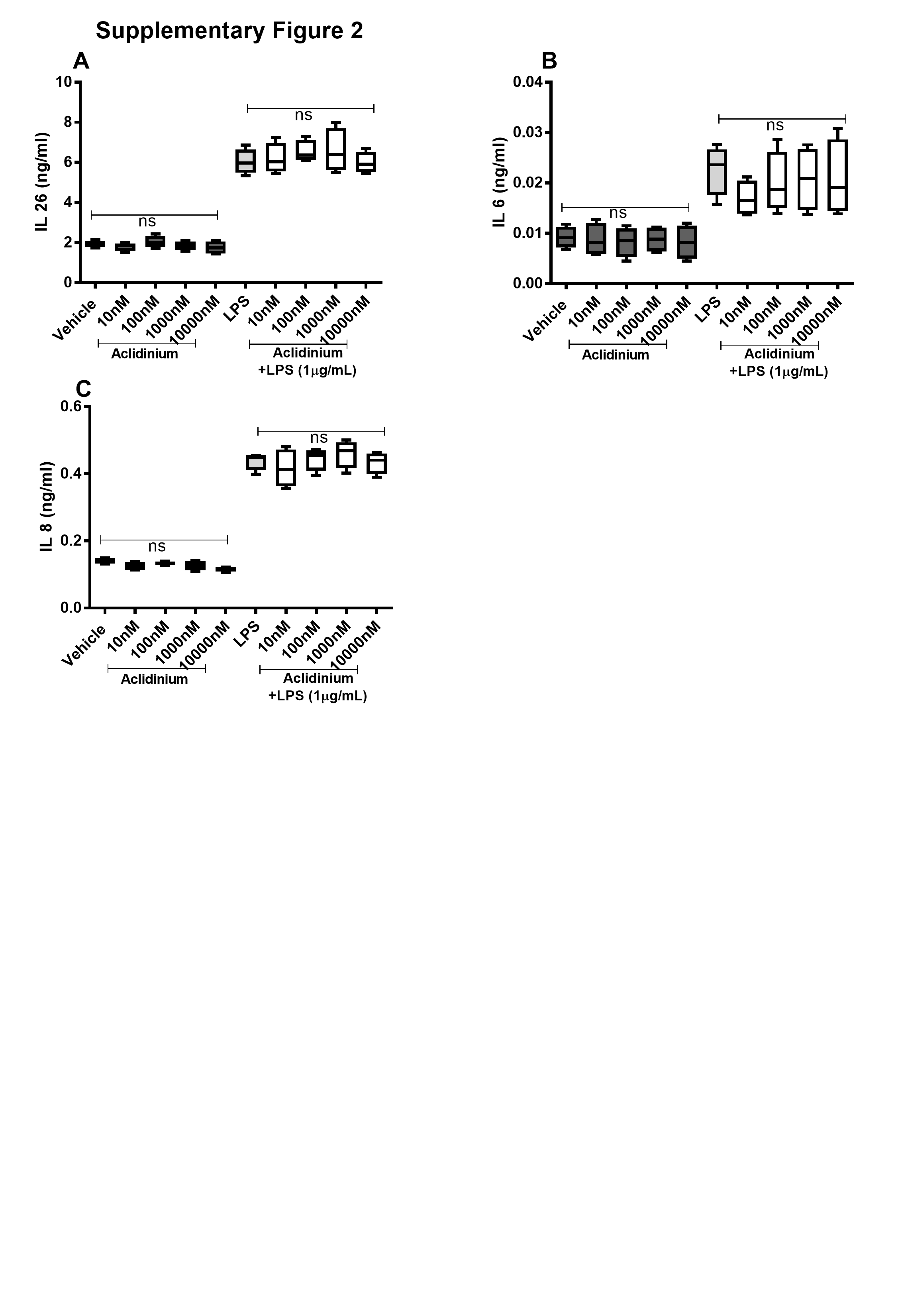

Supplement: Supplementary Figure 2 — Effect of high concentrations of aclidinium on the constitutive and endotoxin-induced release of IL-26, IL-6, and IL-8 in human primary lung fibroblasts. The cells were cultured in vitro and treated with different concentrations of aclidinium with and without endotoxin (lipopolysaccharide, LPS) stimulation (1 µg/mL) for 24 h. The cytokine protein concentrations in cell-free conditioned media were quantified using ELISA. (A) IL-26 concentrations (n = 4). (B) IL-6 concentrations (n = 4). (C) IL-8 concentrations (n = 4). The results are presented as median and range, and the p-values are according to the Mann–Whitney test. p-values < 0.05 were considered statistically significant. [file Image_2.tif]
